# Electronic revolution in plant taxonomy

**DOI:** 10.1186/1471-2148-11-251

**Published:** 2011-09-14

**Authors:** Elizabeth C Moylan

**Affiliations:** 1 BioMed Central, 236 Gray's Inn Road, London, UK

## Abstract

This Editorial highlights recent changes to the International Code of Botanical Nomenclature and the implications these changes have for electronic journals.

## Editorial

The XVIII International Botanical Congress held in Melbourne, Australia this July [1], a gathering of more than 2000 plant scientists from 73 nations, witnessed exciting developments in the field of plant taxonomy. At the meeting, a resolution was passed with a large majority to enable new taxonomic names to be published electronically and their accompanying descriptions and diagnoses to be published in English *or *Latin rather than the obligatory Latin description [2]. These are truly ground sweeping changes to the 'International Code of Botanical Nomenclature' [3] henceforth to be known as the 'International Code of Nomenclature for algae, fungi and plants', and brings 'the Code' bang up to date in the 21^st ^Century.

In her article outlining these changes, Dr Sandra Knapp of the Natural History Museum and Editorial Board Member for *BMC Evolutionary Biology *[4] who chaired the pivotal congress meeting writes with John McNeill and Nicholas Turland on the implications the new code has for scientists, authors and Editors [5].

Previously, online journals including all relevant journals published by BioMed Central [6] faced considerable difficulties when publishing articles which included new taxonomic descriptions. In order to comply with the 'Vienna code' (2005), valid publication was only effected by 'distribution of printed matter to the general public or at least to botanical institutions with libraries accessible to botanists generally' [3]. The code was explicit in stating that valid publication could *not *be effected by' publication online, or by dissemination of distributable electronic media.' Quite a blow for electronic journals who frequently faced common misconceptions from scientists that they simply could not publish online only because they wouldn't be complying with the code.

In order to follow the strictest letter of the code *BMC Evolutionary Biology *and other online journals have had to physically distribute printed copies of relevant articles to appropriate institutions with a relevant explanatory paragraph in the text, see for example, [7,8] and this is also the case for zoological descriptions too [9]. A retrograde step when anyone - wherever they are in the world - can read the articles in question at the click of a button [10].

Thankfully, after an intervening six years, the new 'Melbourne code' is completely transforming for electronic journals. As of the 1^st ^January 2012 it will be possible to publish new taxonomic names online in relevant BioMed Central journals without additional administrative difficulties. While this can only be a positive step for all electronic journals, for *BMC Evolutionary Biology *in particular authors can be reassured that they can easily present the complete picture of their research activities. The molecular and morphological studies we publish often have taxonomic and nomenclatural implications. In the past, these may have been excluded or may have been sent elsewhere. Now, authors will be able to present all these facets in one article without fear that they are not complying with the botanical code. For botanists, *BMC Evolutionary Biology *is truly a home for all aspects of evolutionary research from the molecular to the morphological to the taxonomic and nomenclatural. Let us only hope that the International Code of Zoological Nomenclature for zoologists will soon follow suit [11].
